# Single-session endoscopic ultrasound-guided hepaticogastrostomy and enteral stenting using forward-viewing endoscopic ultrasonography for malignant biliary and duodenal obstruction

**DOI:** 10.1055/a-2239-4822

**Published:** 2024-02-07

**Authors:** Kenji Nakamura, Sakiko Takarabe, Tadashi Katayama, Masataka Ichikawa, Keisuke Ojiro, Hiroshi Kishikawa, Jiro Nishida

**Affiliations:** 1 89421 Department of Gastroenterology, Tokyo Dental College Ichikawa General Hospital, Ichikawa, Japan

Recently, endoscopic biliary and enteral stenting techniques, including the use of endoscopic ultrasound (EUS), have been developed for patients with malignant biliary obstruction (MBO) and malignant gastric outlet obstruction (GOO) 1 2 3 4 . However, when these conditions are encountered simultaneously, including during reintervention, the use of this strategy remains controversial 5 .

Herein, we report the case of a patient with recurrent MBO after biliary stenting and concurrent GOO caused by biliary cancer, managed by EUS-guided hepaticogastrostomy (HGS) and endoscopic enteral stenting using forward-viewing EUS (FV-EUS) in a single session.

An 80-year-old man presented with pyrexia following chemotherapy for biliary cancer. Contrast-enhanced computed tomography (CT) revealed intrahepatic bile duct dilatation ( Fig. 1 ). The diagnosis was acute cholangitis due to stent dysfunction and progressive disease. Emergency biliary drainage failed as the duodenoscope could not pass through the duodenal stenosis caused by the invasive carcinoma. Given the patient’s age, limited life expectancy, and the invasiveness of multiple sedation-requiring endoscopic procedures, we opted for simultaneous EUS-HGS and enteral stenting.

**Fig. 1:**
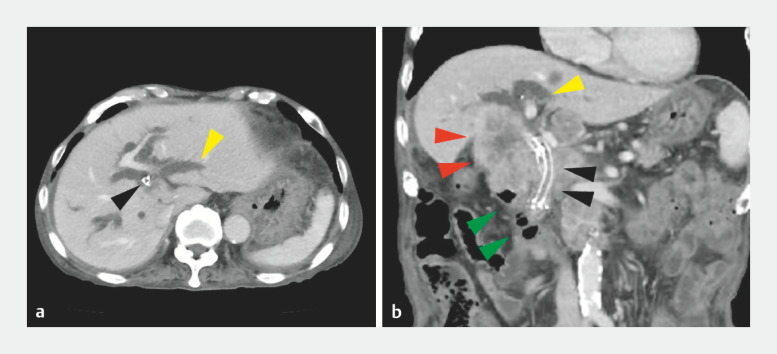
Contrast-enhanced computed tomography (CT) showing dilatation of the bilateral intrahepatic bile duct (B2 branch, yellow arrowheads), a huge mass formed by biliary cancer and metastatic lymph node (red arrowheads), biliary covered metallic stents placed side by side (black arrowheads), and stenosis of the duodenum due to infiltration of the tumor (green arrowheads). **a** Axial view, **b** oblique coronal view.

The dilated intrahepatic bile ducts were confirmed under EUS using FV-EUS (TGF-UC260J; Olympus, Tokyo, Japan) and the left bile duct was punctured with a 19-gauge needle. After injection of contrast medium, a 0.025-inch guidewire was placed. Following dilation of the puncture site using a balloon dilator, the EUS-HGS stent was moved into position over the guidewire under fluoroscopic guidance ( Fig. 2 **a–d** ). Duodenal stenosis was confirmed endoscopically by means of FV-EUS. A guidewire was advanced beyond the stenosis under fluoroscopic guidance to facilitate placement of the enteral stent ( Fig. 2 **e–h** ). Immediately postoperatively, CT confirmed appropriate placement of both the biliary and the enteral stent ( Fig. 3 ). The patient resumed oral intake without complications and was subsequently discharged ( Video 1 ).

**Fig. 2:**
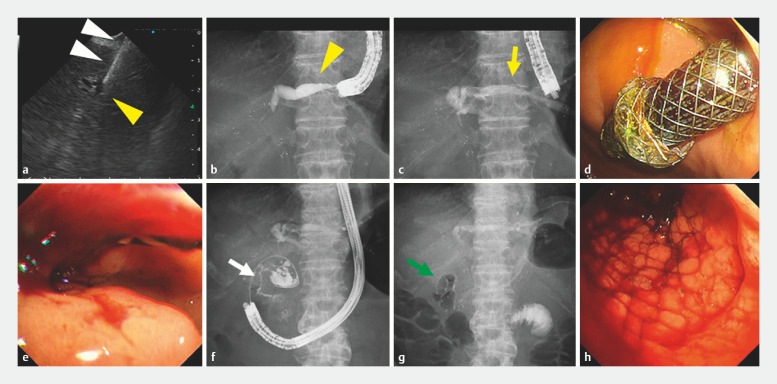
**a** Forward-viewing endoscopic ultrasonography (FV-EUS) showing the left intrahepatic bile duct (B2 branch, yellow arrowhead) slightly dilated and being punctured using a 19-gauge needle (white arrowheads) under EUS guidance. **b** Fluoroscopic view showing confirmation of the B2 branch of the bile duct (yellow arrowhead) injected with contrast medium. **c** Fluoroscopic view showing placement of the EUS-guided hepaticogastrostomy (HGS) stent (yellow arrow). **d** Endoscopic view showing placement of the EUS-HGS stent into the stomach. **e** Conventional endoscopic view of FV-EUS showing the stenosis in the duodenal bulb and placement of the guidewire through the stenosis. **f** Fluoroscopic view showing stenosis (white arrow) from the duodenal bulb to the descending part after injection of contrast medium. **g** Fluoroscopic view showing placement of the enteral stent (green arrow) through the duodenal stenosis. **h** Endoscopic view showing placement of the enteral stent through the duodenal stenosis.

**Fig. 3:**
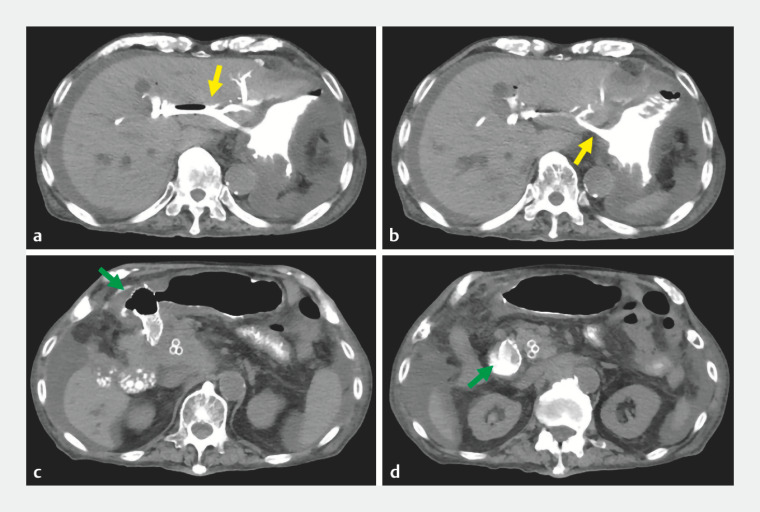
Axial CT immediately after EUS-HGS and enteral stenting: **a, b** EUS-HGS stent (yellow arrows) placed from the B2 branch of the bile duct to the stomach; **c, d** enteral stent (green arrows) placed through the duodenal stenosis caused by the biliary cancer with a metastatic lymph node.

Single-session endoscopic ultrasound-guided hepaticogastrostomy and enteral stenting using forward-viewing endoscopic ultrasonography for malignant biliary and duodenal stenosis.Video 1

This case underlines the potential efficacy of EUS-HGS and enteral stenting in a single session using FV-EUS in high-risk patients, including older adults or those with multiple comorbidities requiring concurrent GOO and MBO management, even in reintervention scenarios. This approach minimizes the need for multiple sedation-requiring endoscopic procedures.

Endoscopy_UCTN_Code_TTT_1AS_2AD

## Notes

Endoscopy E-Videos https://eref.thieme.de/e-videos
*E-Videos* is an open access online section of the journal *Endoscopy* , reporting on interesting cases and new techniques in gastroenterological endoscopy. All papers include a high-quality video and are published with a Creative Commons CC-BY license. Endoscopy E-Videos qualify for HINARI discounts and waivers and eligibility is automatically checked during the submission process. We grant 100% waivers to articles whose corresponding authors are based in Group A countries and 50% waivers to those who are based in Group B countries as classified by Research4Life (see: https://www.research4life.org/access/eligibility/ ). This section has its own submission website at https://mc.manuscriptcentral.com/e-videos .
